# Wind and Wildlife in the Northern Great Plains: Identifying Low-Impact Areas for Wind Development

**DOI:** 10.1371/journal.pone.0041468

**Published:** 2012-07-25

**Authors:** Joseph Fargione, Joseph Kiesecker, M. Jan Slaats, Sarah Olimb

**Affiliations:** 1 The Nature Conservancy, Minneapolis, Minnesota, United States of America; 2 The Nature Conservancy, Fort Collins, Colorado, United States of America; 3 World Wildlife Fund, Bozeman, Montana, United States of America; Trinity College Dublin, Ireland

## Abstract

Wind energy offers the potential to reduce carbon emissions while increasing energy independence and bolstering economic development. However, wind energy has a larger land footprint per Gigawatt (GW) than most other forms of energy production and has known and predicted adverse effects on wildlife. The Northern Great Plains (NGP) is home both to some of the world’s best wind resources and to remaining temperate grasslands, the most converted and least protected ecological system on the planet. Thus, appropriate siting and mitigation of wind development is particularly important in this region. Steering energy development to disturbed lands with low wildlife value rather than placing new developments within large and intact habitats would reduce impacts to wildlife. Goals for wind energy development in the NGP are roughly 30 GW of nameplate capacity by 2030. Our analyses demonstrate that there are large areas where wind development would likely have few additional impacts on wildlife. We estimate there are ∼1,056 GW of potential wind energy available across the NGP on areas likely to have low-impact for biodiversity, over 35 times development goals. New policies and approaches will be required to guide wind energy development to low-impact areas.

## Introduction

**Figure 1:**
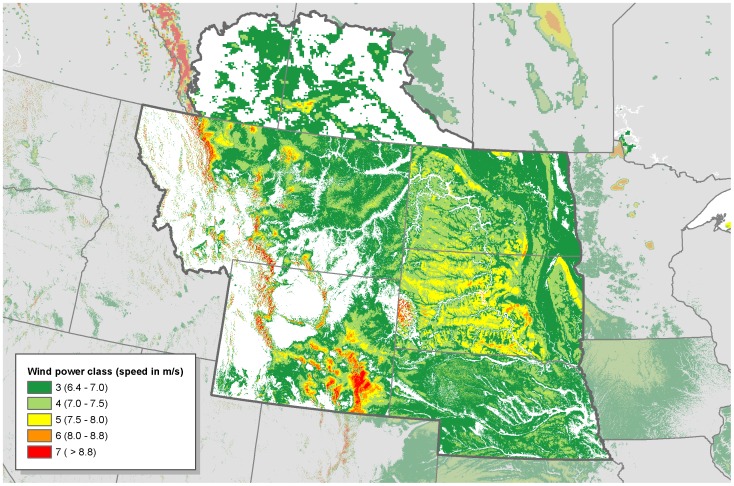
Wind potential in the Northern Great Plains [**30**], [**31**].

The winds on the Northern Great Plains (NGP) are strong and consistent, making this one of the most desirable areas for wind development (Fig. 1). As of January 2012, 5.2 GW of wind energy (all GW numbers refer to nameplate capacity) was in operation in the NGP (including the southern portions of Alberta and Saskatchewan and all of five states: Montana, Nebraska, North Dakota, South Dakota, and Wyoming), and there were 32 GW of proposed wind energy development [1]. The U.S. Department of Energy (DOE) set a goal of producing 20% of U.S. electricity from wind energy by the year 2030 [2]. Nationwide, DOE estimates that this would require 241 GW of on-shore (terrestrial) wind development. In the NGP states, DOE estimates that this would require 25 GW of wind energy. Similar goals in Canada add another 5 GW, for a total of 30 GW of expected development.

Per unit energy, wind energy production requires a much larger area than fossil energy, such that expected wind development is likely to cover large areas of the NGP. The DOE estimates that, with expected continued substantial increases in efficiency, additional capacity will require about 1 km^2^ of land to site 5 MW of wind energy, depending on the quality of the wind resource. Thus, wind energy development is expected to grow to require approximately 5,000 km^2^ across the five United States that compose the NGP. Analogous goals in Alberta and Saskatchewan would require at least 1,000 km^2^ to be developed for wind. It is important to note that the ecological footprint of wind development is likely to be even larger, because many species of wildlife tend to avoid human infrastructure such as wind turbines. For example, sage grouse experience reduced nesting success up to 6.4 km away from oil and gas development [3], [4]. Thus, wind development will impact many thousands of square kilometers in the Northern Great Plains.

Wind energy may have several impacts on wildlife. Direct mortality of birds and bats from wind turbines has been the focus of a growing body of research [5], [6]. Northern Great Plains species of particular concern for direct mortality include whooping cranes (*Grus americana*), golden eagles (*Aquila chrysaetos*), ferruginous hawks (*Buteo regalis*), burrowing owl (*Athene cunicularia*), and several species of bat. Although there is no recorded instance of a whooping crane being killed by a wind turbine, because fewer than 400 individuals remain, any increased mortality would be a significant population concern [7], [8]. Raptors may be particularly vulnerable to wind farm development [9], [10], [11], [12]. Golden eagles are of particular conservation and regulatory concern, due to population declines and their protected status under the Bald and Golden Eagle Protection Act [13], [14]. In places where both bird and bat mortality have been monitored, bat mortality rates can be over an order of magnitude higher than birds, with migratory tree dwelling species being particularly at risk [6], [15].

The large spatial extent of wind development poses conservation concerns in addition to those posed by direct mortality, namely habitat loss and fragmentation. Although direct habitat losses from turbine footings and roads typically comprise less than five percent of a wind energy project area, the habitat values of adjacent lands may be significantly diminished. Habitat loss and fragmentation are considered the most significant threat to threatened and endangered species [16]. Moreover, globally, the temperate grasslands biome is the most converted and least protected [17] and the Northern Great Plains is home to much of North America’s remaining temperate grasslands. In the Northern Great Plains, grassland and shrubland nesting birds, such as the greater sage grouse (*Centrocercus urophasianus*) and the greater prairie-chicken (*Tympanuchus cupido*) are of particular concern because they require large intact areas to maintain viable populations and are likely to avoid use of habitat near roads or turbines [18], [19], [20]. Similarly, large game species depend on crucial summer and winter ranges and migratory corridors that may be impacted by wind and other energy development [21].

Many of the concerns about the impact of wind energy to wildlife can be addressed through appropriate siting. A recent paper suggests that, nationally, over 14 times DOE’s goal for wind energy can be met simply by placing wind turbines on lands that are already disturbed or fragmented [22]. However, this national overview did not attempt to identify the specific locations best suited for low-impact wind development. Here we identify areas in the Northern Great Plains where wind development would have low impact to wildlife by selecting the subset of disturbed lands that have high wind energy potential and are not identified as wildlife priority areas by the best available state-level wildlife data. For each state, we used the best available wildlife and conservation data provided by local and federal agencies and non-profits, focusing on the species, habitats, migration corridors, and stopover sites most likely to be sensitive to wind development. Our approach is not intended to identify all areas suitable for wind development, but rather the subset of areas that, if developed, can be predicted with relative confidence to have low impacts to wildlife.

**Figure 2:**
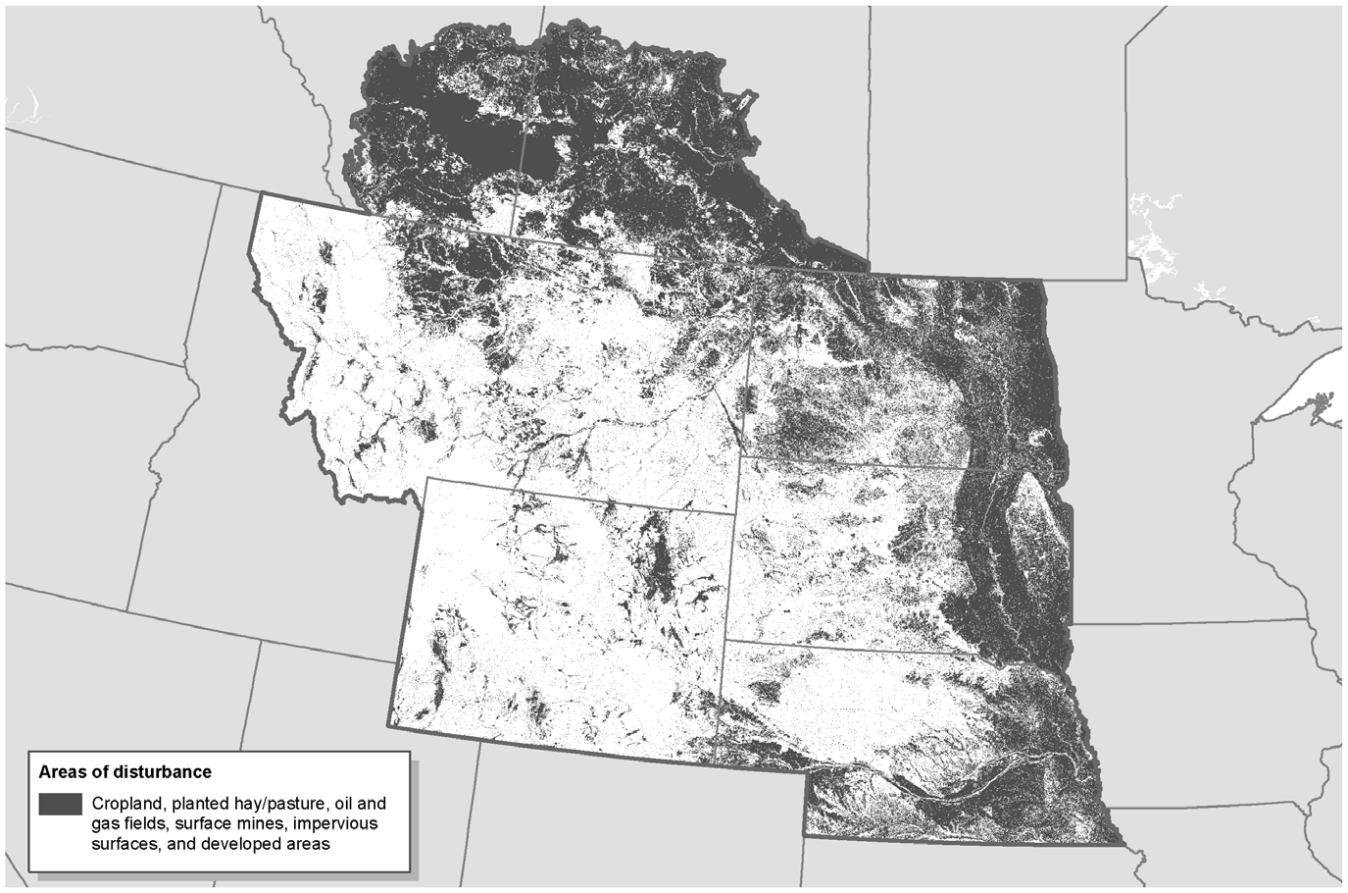
Disturbed areas in the Northern Great Plains [**22]**, [29****].

**Figure 3:**
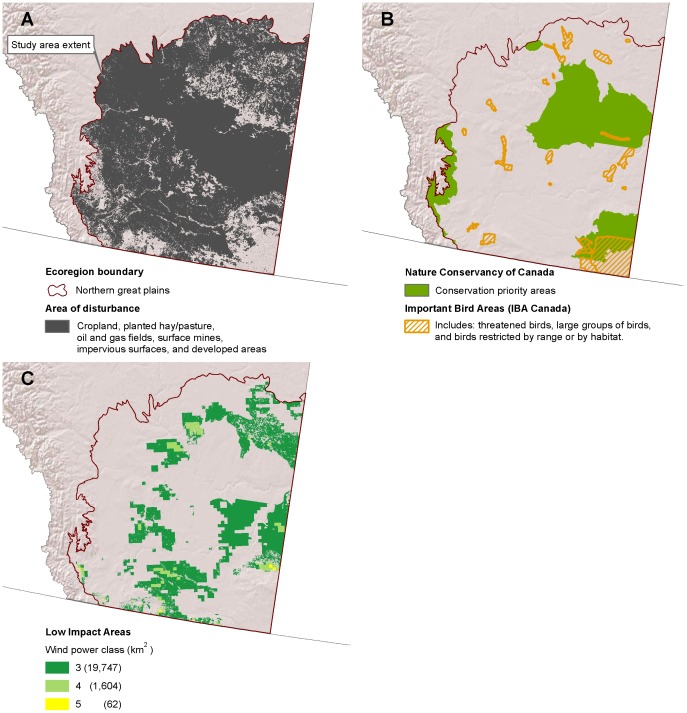
Low-impact areas for wind development in Alberta. ( A) Disturbed areas [29] within the Northern Great Plains ecoregion [23]. (B) Conservation priority areas [40] and important bird areas [38], [39]. (C) Low impact areas for wind development are the subset of disturbed areas where wind is viable and wildlife sensitivity is low.

**Figure 4:**
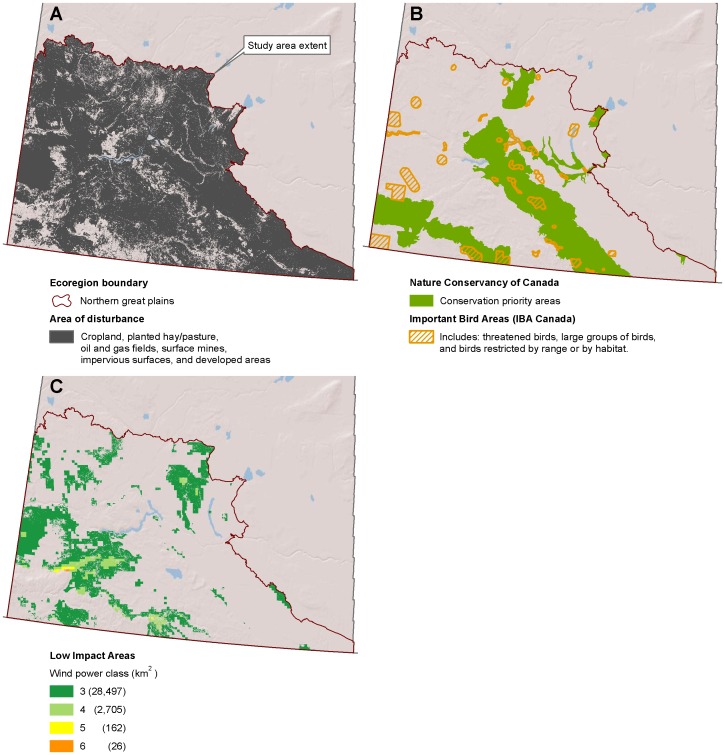
Low-impact areas for wind development in Saskatchewan. ( A) Disturbed areas [29] within the Northern Great Plains ecoregion [24]. (B) Conservation priority areas [40] and important bird areas [38], [39]. (C) Low impact areas for wind development are the subset of disturbed areas where wind is viable and wildlife sensitivity is low.

**Figure 5:**
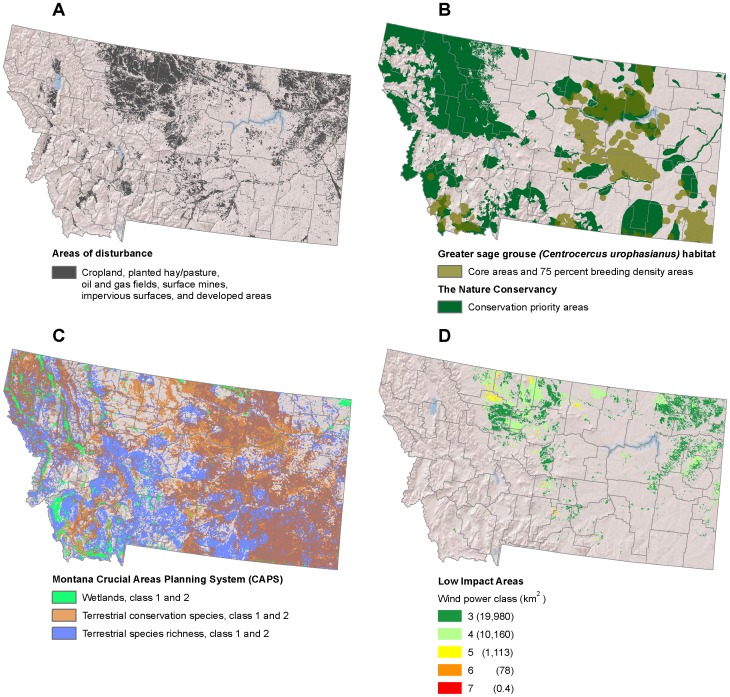
Low-impact areas for wind development in Montana. (A) Disturbed areas [22]. (B) Greater sage grouse (*Centrocercus urophasianus*) habitat [42], [43] and conservation priority areas [44]. (C) Montana Crucial Areas Planning System priority areas for wetlands, terrestrial species, and terrestrial species richness [41]. (D) Low impact areas for wind development are the subset of disturbed areas where wind is viable and wildlife sensitivity is low.

**Figure 6:**
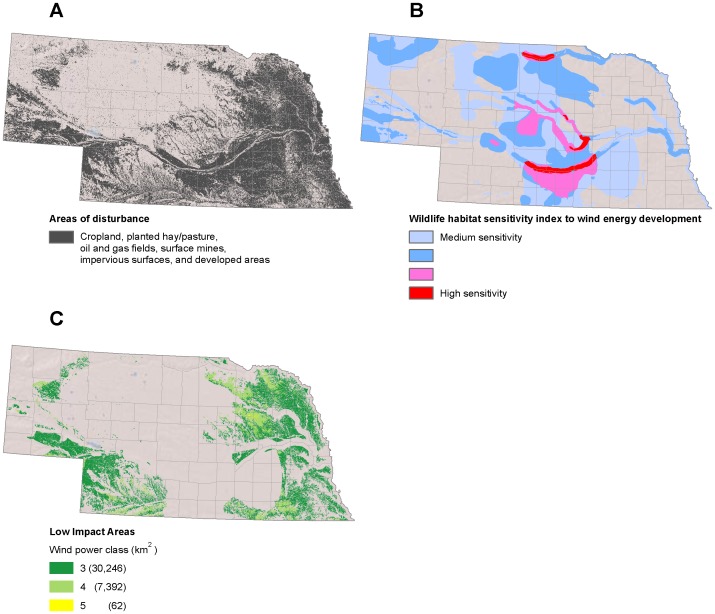
Low-impact areas for wind development in Nebraska. ( A) Disturbed areas [22]. (B) Areas with medium to high wildlife sensitivity to wind energy development [45]. (C) Low impact areas for wind development are the subset of disturbed areas where wind is viable and wildlife sensitivity is low.

**Figure 7:**
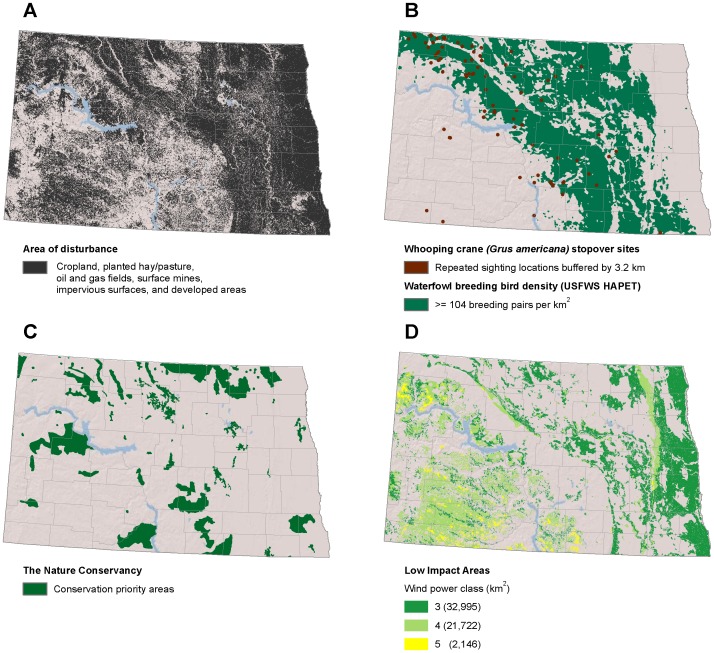
Low-impact areas for wind development in North Dakota. (A) Disturbed areas [22]. (B) Whooping crane (*Grus americana*) stopover sites and waterfowl breeding bird density [47], [48]. (C) conservation priority areas [44]. (D) Low impact areas for wind development are the subset of disturbed areas where wind is viable and wildlife sensitivity is low.

**Figure 8:**
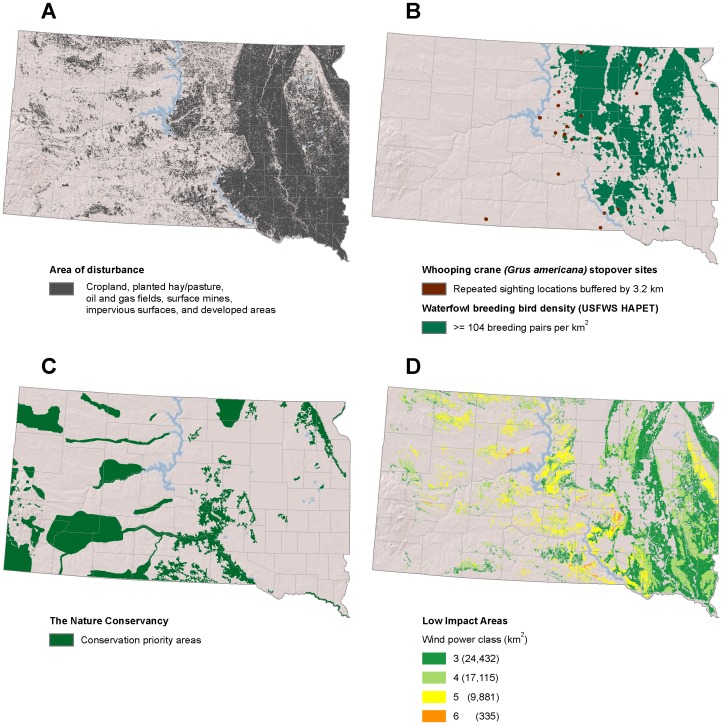
Low-impact areas for wind development in South Dakota. (A) Disturbed areas [22]. (B) Whooping crane (*Grus americana*) stopover sites and waterfowl breeding bird density [47], [48]. (C) conservation priority areas [44]. (D) Low impact areas for wind development are the subset of disturbed areas where wind is viable and wildlife sensitivity is low.

**Figure 9:**
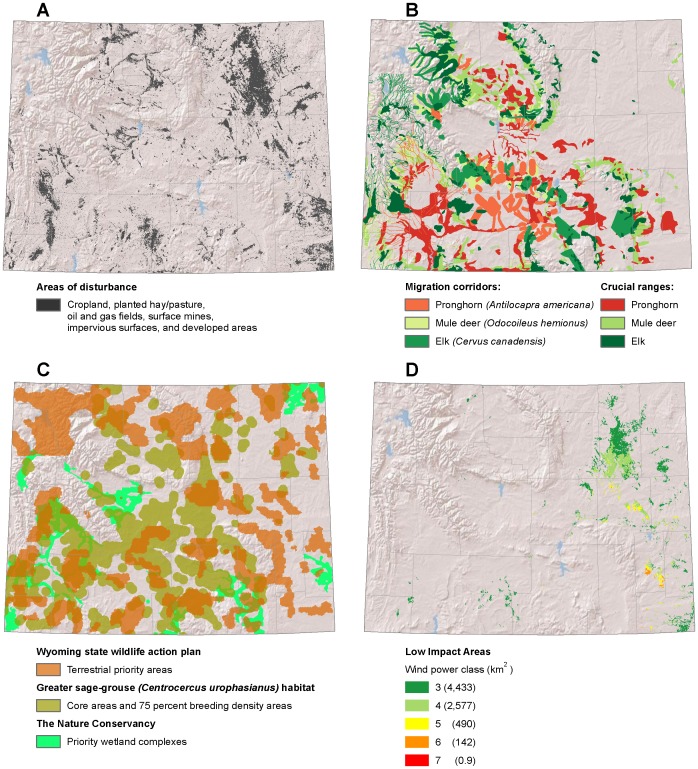
Low-impact areas for wind development in Wyoming. (A) Disturbed areas [22]. (B) Ungulate migration corridors and crucial ranges [51]. (C) Wyoming state wildlife action plan terrestrial priority areas [50], Greater sage grouse (*Centrocercus urophasianus*) core areas [42], [52], and priority wetland complexes [53]. (D) Low impact areas for wind development are the subset of disturbed areas where wind is viable and wildlife sensitivity is low.

**Figure 10:**
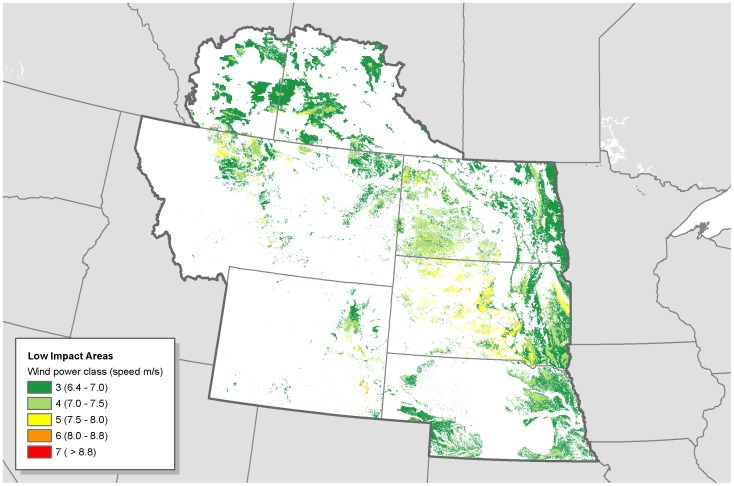
Low-impact areas for wind development in the Northern Great Plains. Low impact areas for wind development are the subset of disturbed areas where wind is viable and wildlife sensitivity is low.

## Methods

Based on data availability, we defined a study area based on political boundaries in the United States and ecological boundaries in Canada. Our Canadian analysis was constrained to the Northern Great Plains ecoregion, defined to include Northern mixed and short grassland ecoregions [23], [24].

### Disturbance Data

We created a binary disturbed/undisturbed classification that considers areas with any of several human impacts to be disturbed. Specifically, we compiled spatial data on the footprint of human disturbance, including developed areas, cropland, roads and other impervious surfaces, oil and gas development, and surface mines.

For the United States, we used the disturbance data compiled by Jeffrey Evans [22]. This compilation includes data from four sources: 1) 2001 National Land Cover Dataset, 2) USGS impervious surfaces dataset, 3) USGS topographic change dataset, and 4) a national oil and gas field dataset. The National Land Cover Dataset (NLCD) is a federal multi-agency effort that applies standard class schemas, methodologies, and error assessments to quantify land cover patterns across the entire lower 48 United States [25]. The following NLCD classes were considered to be disturbed lands: Cultivated Crops, Developed-High Intensity, Developed-Low Intensity, Developed-Medium Intensity, Developed-Open Space, and Hay/Pasture. The Hay/Pasture class included planted forage grasses, but did not include natural (i.e. unplanted) grasslands used for grazing. We used a Landsat derived impervious surface classification produced by USGS [26] to identify areas with reduced percolation such as pavement. The USGS topographic change data was used to identify significant topographic change, representing surface mines and other major human-based changes in topography [27]. The USGS used a threshold for identifying significant topographic change of 10.21–17.57 meters, depending on the land cover type. Oil and gas fields were identified with a kernel density analysis of well locations using IHS energy^©^ data [28].

We compiled disturbance data for Canada, including the following datasets: 1) A land cover database from Agriculture and Agri-Food Canada, classified circa 2000 Landsat imagery [29]. The following land cover classes were considered to be disturbed lands: Developed, Cultivated Agricultural Land, Annual Cropland, Perennial Cropland, and Pasture; 2) Active oil and gas fields based on a kernel density analysis of existing oil and gas wells; 3) Pipelines, roads, railways and transmission lines, rasterized at 30 meters. To identify urban areas, we used data on hamlets, villages, towns and cities. We converted point data on hamlets to raster data by buffering each hamlet by 300 meters and rasterizing at 30 meters.

### Wind Resource and Development Goals

For wind power in the United States, we used wind power class modeled at 50 meters above the ground [30]. These data are available at a resolution of 200×200 meter pixels. For wind power in Canada, we used wind power class at 50 meters above the ground as estimated in the Canadian Wind Energy Atlas [31]. These data are available at a resolution of 5 km x 5 km pixels. We considered wind power class 3 and higher (≥6.4 m/s) to be economically viable [2]. Although newer wind turbines commonly have a hub height of 80–100 meters, wind speed data at this height is not publically available as a GIS data layer. Since wind speed increases with height, our use of the 50-meter wind speed data is conservative in two respects. First, all of the areas that we identify as low-impact areas for development have wind power classes as good or better than assumed for our analysis. Second, our estimates of the amount of low-impact areas available for wind development are conservative, because some areas that our analysis considers to have insufficient wind resource may actually be viable for development.

We obtained wind development goals for U.S. states from the DOE [2]. We are not aware of comparable governmental goals for southern Alberta or Saskatchewan. Therefore we used the amount of proposed wind energy development in the connection queue of the Alberta Electric System Operator as of December 2011 [32] as an estimate of the magnitude of additional wind development in Alberta that could be targeted to low-impact areas. For Saskatchewan, we used a wind development goal of 20% of the projected total nameplate capacity for all electricity generation by 2030 [33].

### State-Level Wildlife Data

The disturbed areas used in this analysis represent low-quality habitats that we expect are altered to the point that they no longer support natural community assemblages or populations of species of conservation concern [34]. Disturbance is consistently associated with increased probability of extirpation for many species, such that areas of high disturbance generally have low value for biodiversity [34], [35], [36], [37].We recognize, however, the limitations of our disturbance dataset to fully capture and represent local-scale biodiversity values. To more fully capture biodiversity values within each state/province, we assembled local data layers that identify areas with significant conservation values within each state. These areas were then excluded from designation as low-impact areas for wind development, even if they were already disturbed. We obtained the best available state-level data for wildlife priority areas in Montana, Nebraska, North Dakota, South Dakota, Wyoming, and southern Alberta and Saskatchewan.

### Alberta and Saskatchewan

Alberta and Saskatchewan had similar species of concern and data availability, allowing a consistent methodology across these provinces. We excluded Important Bird Areas in Canada [38]. Important Bird Areas in Canada are home to threatened birds, large groups of birds, and birds restricted by range, and were identified as a part of an international effort to identify important bird areas using consistent criteria [39]. We also excluded Priority Natural Areas identified by the Nature Conservancy of Canada to represent the best examples of the diversity of the provinces’ natural heritage and key habitat for many species [40].

### Montana

Montana Fish, Wildlife & Parks has created a Crucial Areas Planning System to identify areas that may be relatively more sensitive to development [41]. They generated several data layers, including a terrestrial species richness layer, a terrestrial conservation species layer, and a wetlands layer. Each data layer was categorized into four classes. For each of these three data layers we excluded the two categories of highest conservation concern. For sage grouse, we excluded both the range-wide 75% core breeding areas [42] and Montana core areas map [43]. We also excluded The Nature Conservancy’s conservation priority areas [44].

### Nebraska

The Nebraska Game and Parks Commission has conducted a wind and wildlife analysis that identifies areas of relative sensitivity for species of conservation concern that may be impacted by wind energy development [45]. Their analysis considered the following species: bald eagle (*Haliaeetus leucocephalus*), golden eagle (*Aquila chrysaetos*), bighorn sheep (*Ovis canadensis*), ferruginous hawk (*Buteo regalis*), greater prairie-chicken (*Tympanuchus cupido*), interior least tern (*Sternula antillarum athalassos*), long-billed curlew (*Numenius americanus*), mountain plover (*Charadrius montanus*), piping plover (*Charadrius melodus*), sharp-tailed grouse (*Tympanuchus phasianellus*), whooping crane (*Grus americana*), and three species of bats. Their analysis ranked the relative sensitivity of all areas within the state of Nebraska on a scale of 1–6. To identify low-impact areas for wildlife, we excluded the areas within the four most sensitive categories. We also excluded playa lakes identified as high or very high quality by the Playa Lakes Joint Venture [46].

### North Dakota and South Dakota

North Dakota and South Dakota had similar species of concern and data availability, allowing a consistent methodology across these states. We excluded areas with a predicted waterfowl breeding pair density of 104 pairs per km^2^ (40 pairs per mile^2^) or greater, based on US Fish and Wildlife Service Habitat And Population Evaluation Team data [47], [48]. This represents crucial habitat for many species of wetland-dependent birds in North America [47], [48]. We excluded The Nature Conservancy’s conservation priority areas [44]. We also excluded repeated whooping crane stopover sites, buffered by 3.2 km [49]. To identify repeated whooping crane stopover sites, we included any site with whooping crane observations in multiple years or on three or more days in the same year, based on U.S. Fish and Wildlife Service observation data. We also excluded a 1.6 km buffer area around the Missouri and Red Rivers that are important migratory corridors for birds and bats.

### Wyoming

We excluded wildlife priority areas identified by the Wyoming State Wildlife Action Plan [50]. For big game ungulates (pronghorn, *Antilocapra americana*; elk, *Cervus canadensis*; and mule deer, *Odocoileus hemionus*) we excluded migration corridors and crucial winter and summer ranges [51]. For sage grouse, we excluded both the range-wide 75% core breeding areas [42] and the Wyoming Governor’s core areas [52]. We also excluded priority wetland complexes [53].

### Analysis

We identified lands potentially suitable for wind development by excluding lands known to be unsuitable for wind development based on wind speeds, protected status, urbanization, or standing water. We excluded lands with average annual wind speeds of less than 6.4 meters per second (i.e., wind power classes 1 and 2 in the NREL data). We excluded protected areas where wind development would likely be prohibited based on areas with a Gap Analysis Program code 1 or 2 (i.e., permanent protection that excludes development), based on the Protected Area Database of the United States [54]. We used updated protected areas data for Wyoming [55] and Montana [56]. We excluded urban-core areas [57]. We also excluded wetlands and water bodies identified in the NLCD. To exclude disturbed areas that are too small to support wind development, we removed all patches of disturbed areas that were smaller than 1 km^2^ and greater than 800 m away from patches of disturbed areas greater than 1 km^2^.

We quantified the GW of wind energy in each state/province that could be produced on suitable low-impact disturbed lands. We accounted for the fact that areas with higher wind speeds will generate more electricity on the same amount of land as follows. We estimated the amount of MW per unit area, following DOE [2], by assuming that projected average nameplate capacity (44.5%) is installed at 5 MW/km^2^ and adjusting turbine nameplate capacity based on capacity factors specific to each wind power class. The relationship between production capacity and area is predicted to vary by wind power class as follows: WPC 3 = 4.3 MW/km^2^; WPC 4 = 4.8 MW/km^2^; WPC 5 = 5.2 MW/km^2^; WPC 6 = 5.5 MW/km^2^; WPC 7 = 6.0 MW/km^2^. In addition, we evaluated whether wind turbines, current and proposed, as of early 2012 [1], [58] are inside or outside of the low impact disturbed lands that we identified.

## Results

**Figure 11:**
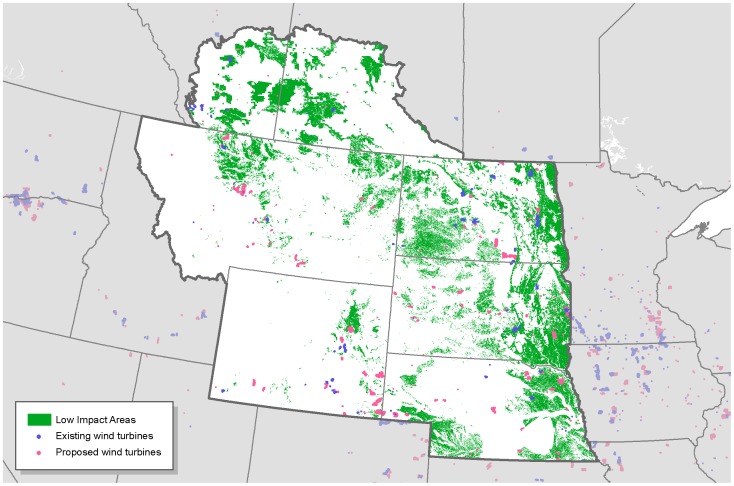
Current and proposed wind turbine locations in relation to low-impact areas in the Northern Great Plains.

Disturbed areas in the study region comprised 447,778 km^2^ (Figure 2). We find that there are large areas where wind could be prioritized that would likely have little impact to wildlife (Figures 3, 4, 5, 6, 7, 8, 9 and 10). Across all states/provinces in the Northern Great Plains, there is enough wind resource on low-impact disturbed land to produce over 35 times the projected wind development goals (Table 1). Most (78%) of the low-impact lands that we identified were cropland. Current wind turbines in the Northern Great Plains are sited within our identified low impact areas 34% of the time, compared with 30% of the time for proposed turbines (Figure 11; Table 2).

**Table 1:** Wind energy production capacity on disturbed lands with low predicted impact to wildlife in the Northern Great Plains.

State	GW Goal	GW on low-impact lands	% of DOE goal
Alberta	4.11	91	2215%
Montana	5.26	139	2643%
Nebraska	7.88	163	2069%
North Dakota	2.26	254	11239%
Saskatchewan	1.10	137	12455%
South Dakota	8.06	238	2953%
Wyoming	1.28	34	2662%
Total	29.95	1,056	3526%

**Table 2:** Distribution of current and proposed wind turbines in the Northern Great Plains in relationship to low-impact disturbed areas.

	Existing			Proposed		
State	# wind turbines	# Low impact wind turbines	% low impact	# wind turbines	# Low impact wind turbines	% low impact
Alberta	312	73	23%	1413	632	45%
Montana	264	128	48%	998	260	26%
Nebraska	148	52	35%	1208	500	41%
North Dakota	885	392	44%	1085	233	21%
South Dakota	351	147	42%	892	442	50%
Saskatchewan	115	100	87%	189	16	8%
Wyoming	571	1	0%	1316	41	3%
Total	2646	893	34%	7101	2124	30%

## Discussion

Our results indicate that there is ample opportunity in the Northern Great Plains (NGP) to target wind development to lands that are already disturbed and would likely have low impact to wildlife. We estimate that 1,056 GW of wind energy could be produced on these low-impact lands. This is over 35 times the goal for wind energy development in this region. The majority of the low-impact areas that we identified were located on cropland, indicating the benefit of co-locating wind facilities on existing cropland. The fact that the availability of wind energy far exceeds development goals indicates that our results are not dependent on the particular development goals that we have used, but would be applicable for any reasonable goal.

We recommend that wind energy be targeted toward these areas with relatively low impact to wildlife. We recognize that wildlife impacts are only one consideration associated with wind energy development, and we have not attempted to address other issues, such as areas of cultural significance, in this analysis. We note that this analysis is not intended to be exhaustive, and that there are places outside of the areas that we have identified where low-impact wind development is possible. Additional research is necessary to identify these areas. Similarly, even within the areas we have identified, there may be wildlife resources that need to be avoided through proper micro-siting and best management practices, which may include curtailment of wind turbines at low wind speeds to reduce bat mortality [59]. While it is clear that wind turbines have little additional impact on habitat and fragmentation in places that are already converted and fragmented, the relationship between disturbance and direct mortality is less clear. Numerous studies have noted that bird species of conservation concern are less abundant in areas that are converted and fragmented, suggesting that mortality for species of conservation concern are likely to be lower in disturbed areas [34]. However, additional research is needed to quantify the relationship between bird and bat mortality and landscape features, including land cover/uses such as croplands and oil and gas fields. Finally, we note that these recommendations are based on the best available wildlife data, but for some species these data are poor. For example, additional data on the migratory patterns of at-risk bat and bird species would be particularly useful for refining our recommendations.

Our analysis finds that the majority (70%) of proposed development in the Northern Great Plains is outside of the low-impact areas we have identified, suggesting that the current regulatory framework is generally insufficient to ensure low-impact wind development. Currently, conscientious developers who avoid a site that has substantial wildlife impacts may be at a competitive disadvantage because a competitor could subsequently develop the site. Consequently, relying on individual developers to voluntarily improve siting practices is unlikely to achieve desired conservation outcomes, because sensitive areas avoided by one project can be easily impacted by subsequent development. Also unlikely, for political reasons, is significant additional regulation in the NGP that restricts the development of wind resources on private lands based on wildlife concerns. Rather, improved incentives such that conscientious developers receive a competitive advantage will likely be necessary for widespread adoption of wildlife-friendly development practices. We identify four areas where action to help change incentives is needed: 1) transmission line siting; 2) formal guidelines and certification; 3) utility power purchase decisions; and 4) appropriate compensatory offsite mitigation for unavoidable impacts.

Wind development is limited by the availability of transmission to bring generated power to market. Consequently, the development of new transmission lines can strongly influence where new wind energy facilities will be developed. We suggest that transmission line siting target the disturbed, low-impact areas identified in this analysis.

Formal guidelines and certification would benefit industry by providing transparent guidelines that reduce risks for developers. Environmental issues can cause costly project delays or abandonment. Avoiding such costs could serve as an incentive for voluntary adoption of guidelines by wind project developers. Perhaps more importantly, compliance with guidelines or certification could serve as a basis for power purchase decisions by utilities. Utilities could indicate in their requests for proposals for new power generation that projects meeting wildlife guidelines would be given preference. Because wind development typically cannot be financed without a long term power purchase agreement, this would provide strong incentive for wind developers to comply with any guidelines so endorsed. Such guidelines should not only identify low-impact areas, but should also identify avoidance areas where wind development could not be certified. In addition to low-impact and avoidance areas, there are intermediate areas that would incur moderate impacts that could be mitigated with compensatory offsite mitigation [60]. For example, impacts to moderately fragmented grasslands could be offset with grassland restoration or with protection of existing intact grasslands. Our work provides a substantial starting point for such conservation strategies, compiling many of the data layers that would be needed to construct comprehensive guidelines including avoidance areas and mitigation costs. These data are available at http://LowImpactWind.tnc.org.

### Conclusion

In places where extensive wind development may conflict with the preservation of large intact landscapes and the wildlife that depend upon them, strategies that can balance the needs of development and conservation are required. Targeting wind energy development to areas with low impacts to wildlife can help society simultaneously achieve goals for clean renewable energy production and wildlife conservation. Encouragingly, our analysis demonstrates that is possible to meet all of our demand for wind energy on lands that are likely to have low impacts to wildlife. In the Northern Great Plains, wind energy production on low-impact disturbed lands could exceed 1,056 GW, over 35 times the projected demand for wind energy. New policies and approaches are needed to guide wind energy development to low impact areas.
